# EDIN and N-PASS pain scale comparison in asphyxiated newborns treated with therapeutic hypothermia

**DOI:** 10.3389/fpain.2026.1783611

**Published:** 2026-03-23

**Authors:** Licia Lugli, Elisabetta Garetti, Maria Federica Roversi, Arianna Bianchini, Riccardo Cuoghi Costantini, Isotta Guidotti, Michele De Novellis, Eugenio Spaggiari, Paola Lago, Alberto Berardi

**Affiliations:** 1 Neonatology and Neonatal Intensive Care Unit, University Hospital of Modena, Modena, Italy; 2 Statistic Unit, University Hospital of Modena, Modena, Italy; 3 Neonatology Unit, Cà Foncello Hospital Treviso, Treviso, Italy

**Keywords:** asphyxia, EDIN, N-PASS, pain, therapeutic hypothermia

## Abstract

**Background:**

Therapeutic hypothermia (TH) is the standard treatment for moderate to severe hypoxic–ischemic encephalopathy (HIE), yet pain assessment during TH remains challenging. This study compares two validated pain scales in asphyxiated newborns undergoing TH and receiving fentanyl analgesia.

**Methods:**

Twenty term infants with HIE treated with TH were enrolled. Pain was assessed using EDIN and N-PASS, while sedation was monitored using the N-PASS sedation subscale.

**Results:**

N-PASS pain and sedation scores significantly decreased by day 3, whereas EDIN scores showed no significant temporal change. Effective analgesia significantly increased over time, either when defined based on EDIN (OR 3.12, *p* = 0.002) and on N-PASS (OR 3.06, *p* = 0.007), while N-PASS sedation did not show a time-dependent association. No scale showed a significant association with fentanyl dosage. A moderate positive correlation was found between EDIN and N-PASS pain scores (*r* = 0.409, *p* < 0.001). Sedation targets were achieved in only 40%–50% of assessments, with early undersedation and later oversedation observed.

**Conclusions:**

EDIN and N-PASS pain scores demonstrate moderate concordance, but capture different dimensions of neonatal pain during TH. N-PASS appears more sensitive to temporal changes, likely due to its combined behavioral and physiological components, whereas EDIN may be affected by hypothermia-related behavioral suppression.

## Highlights

- This study compares the EDIN and N-PASS pain scales in asphyxiated newborns undergoing therapeutic hypothermia, where analgesia is particularly challenging.
- Pain and sedation assessments showed different temporal patterns, with N-PASS more responsive to change than EDIN, while the two scales still demonstrated a moderate correlation.
- Findings underscore the difficulty of maintaining stable analgesia and sedation during therapeutic hypothermia and the need for improved assessment tools in this context.

## Introduction

Neonatal encephalopathy resulting from perinatal asphyxia remains one of the leading causes of neonatal mortality and long-term neurological morbidity worldwide (1–4). Over the past two decades, therapeutic hypothermia (TH) has become the standard of care for term newborns with moderate to severe hypoxic–ischemic encephalopathy (HIE)**.** Robust evidence from randomized controlled trials and meta-analyses has shown that TH significantly reduces mortality and the risk of severe neurodevelopmental impairment (1–8). Despite its neuroprotective benefits, TH is associated with several physiological and clinical challenges (9–13). During the cooling phase, newborns may experience stress, discomfort, and pain**,** which can negatively affect hemodynamic stability, metabolic balance, and potentially the overall therapeutic efficacy of hypothermia. Moreover, untreated pain or stress may trigger adverse neuroendocrine responses, whereas excessive use of sedatives and analgesics to control discomfort may also carry potential risks for the developing brain. Accurate pain assessment therefore plays a crucial role in the management of infants undergoing TH (9–13). However, pain evaluation in neonates with HIE is particularly complex. Neurological depression, sedation, and therapeutic hypothermia itself can affect behavioral and physiological responses, making standard pain assessment less reliable. For this reason, several validated neonatal pain scales have been developed and applied in clinical practice, each with definite strengths and limitations, none of them developed specifically for TH (9–13). There is currently no consensus regarding which scale provides the most accurate and clinically useful assessment of pain in neonates undergoing therapeutic hypothermia for HIE. Comparative studies are limited, and the reliability of some tools may be affected by the altered neurological and physiological state of these infants. For this reason, the present study aimed to compare two neonatal pain assessment scales—the Neonatal Pain, Agitation and Sedation Scale (N-PASS) and the Échelle Douleur Inconfort Nouveau-Né (EDIN)**—**in the specific context of TH for HIE (14–18). We hypothesized that although EDIN and N-PASS scores would be correlated, the multidimensional N-PASS scale—which incorporates physiological parameters—would prove to be a superior and more clinically useful tool for monitoring both pain and sedation in neonates undergoing hypothermia therapy. By evaluating their consistency and performance over time, we aimed to contribute to the identification of the most appropriate tool for optimal pain management in this vulnerable population.

## Methods

### Patients

Newborns affected by moderate to severe HIE and treated with TH at the NICU of the University Hospital of Modena since 1 January 2022 to December 2024 were eligible for this prospective cohort study. The selection of patients for TH was based on previously established criteria: (1) gestational age ≥ 37 weeks; (2) intrapartum asphyxia, defined by at least one of the following: an Apgar score ≤ 5 at 10 min, ventilation with an endotracheal tube or a mask for at least 10 min, or metabolic acidosis within 60 min of birth (cord pH or any arterial/venous pH ≤ 7.0 or base defect ≥ 12 mmol/L); (3) neonatal encephalopathy evaluated within 1 h of birth according to Sarnat modified criteria; and (4) moderate to severe EEG polygraphic (pEEG) or amplitude-integrated EEG (aEEG) abnormalities (3, 4, 13, 19–22). Other exclusion criteria were congenital malformations, chromosomal abnormalities, either suspected or confirmed metabolic disorders, sepsis or central nervous system infections, and different causes of asphyxia (i.e., sudden, unexpected postnatal collapse). Patients were cooled to a rectal temperature of 33.5 °C for 72 h (CritiCool MTRE, Charter Kontron, Milton Keynes, UK). After 72 h of cooling, slow, controlled, and monitored rewarming was started, increasing the set temperature by 0.5 °C every hour. In this way, the patient achieved a 36 °C core temperature in about 5–6 h. If any complication occurred during this period (i.e., seizure), rewarming was transiently interrupted and restarted when clinically indicated (3, 4, 13, 19–21). Every enrolled patient was administered an indwelling double-lumen umbilical catheter with one of the two lumina dedicated to fentanyl infusion. Enrolled newborns received a fentanyl loading dose of 2 mcg/kg as an intravenous bolus (in 20 min), which was immediately followed by 1 mcg/kg/h as a continuous infusion during TH and the rewarming phase. The continuous fentanyl infusion could be increased by 25%–50% if discomfort or pain arose. Shivering, a 20% increase in heart rate over the baseline, facial grimaces, and EDIN score > 6 or N-PASS scores >3 were considered signs of discomfort or pain (13, 22).

### Pain measurement

Pain assessments were performed by the bedside nurses (BNs) responsible for the clinical care of each infant. All participating BNs held specialized qualifications in neonatal intensive care. Prior to the initiation of the study, the entire nursing staff of the unit underwent a standardized, structured training program on the administration of both the EDIN and N-PASS scales. This training consisted of a theoretical session reviewing the scoring criteria for each item on both scales, followed by a practical calibration phase. During the calibration phase, nurses independently scored a series of standardized video recordings of neonates (not included in the study) exhibiting varying states of pain, agitation, and sedation. Their scores were then compared against a pre-determined gold-standard scoring provided by two senior specialists with over 10 years of NICU experience and certified expertise in neonatal pain assessment. Discrepancies were discussed in a group session to reach a consensus on correct application, with the goal of maximizing inter-rater agreement. To maintain consistency throughout the data collection period, refresher training sessions were held quarterly. EDIN and N-PASS scores were recorded simultaneously three times daily (T1, T2, T3) during hypothermia and rewarming, coinciding with the nursing staff's routine assessment schedule (13). It should be noted that while active cooling lasted 72 h (Days 1–3), “Day 4” in this analysis refers to the period of rewarming, during which infants became normothermic but remained critically ill in the NICU. EDIN is a unidimensional validated scale using five behavioural indicators of prolonged pain: facial activity, body movements, quality of sleep, quality of contact with nurses, and consolability (18). Each indicator is scored from 0 to 3**,** resulting in a total score ranging from 0 to 15**.** A score greater than 6 is generally considered indicative of the presence of pain**,** warranting clinical attention and potential intervention (13, 18). N-PASS algometric scale encompasses five indicators: (1) crying/irritability (with a silent cry observed in intubated infants scored as a cry), (2) behavioral state, (3) facial expression, (4) extremities/tone, and (5) vital signs. The criteria are assigned values of 0, 1, or 2 (for pain/agitation) and 0, −1, or −2 (for sedation) (14–17). The N-PASS pain scores range from 0 to 10 for term newborns. A N-PASS score >3 indicates moderate pain. The N-PASS score for sedation ranges from 0 to −10 (moderate sedation: an N-PASS score between −2 and −5; profound sedation: an N-PASS score between −5 and −10) (14–17). In this study, the targeted level of sedation was moderate sedation (N-PASS score between −2 and −5).

To standardize the context of assessment as much as possible within clinical constraints, the following protocol was established:

1. N-PASS pain score and EDIN score: these assessments were conducted while the infant was at rest, ideally at least 30 min after a scheduled caregiving intervention (e.g., diaper change, suctioning, repositioning) or a painful procedure. The bedside nurse observed the infant for a full 2-minute period without direct stimulation before assigning scores. This approach aimed to capture a “baseline” level of prolonged discomfort or pain, as intended by these scales.
2. N-PASS sedation score: In contrast, the N-PASS sedation subscale was evaluated specifically during and immediately after routine nursing maneuvers, such as gentle handling for assessment or diaper change. This approach is aligned with the scale's validation, as responsiveness to handling is a key behavioral indicator of sedation depth. The score reflected the infant's lowest level of responsiveness observed during the maneuver.

This protocol was designed to ensure that pain/discomfort scores were not artificially inflated by procedural stress, while sedation scores were evaluated in a context that would elicit a behavioral response, thereby providing a more accurate and clinically relevant assessment of each construct.

Pain and sedation assessments (T1, T2, T3) continued for the rewarming period (T1 in Day 4) to capture the immediate post-hypothermia phase.

For the purpose of this study, we defined clinical outcomes based on established thresholds for each scale:

1. Effective analgesia: a state where the pain score was at or below the recognized threshold for clinically significant pain. This was defined as an EDIN score ≤ 6 or an N-PASS pain score ≤ 3.
2. Ineffective Analgesia/Presence of Pain: an EDIN score > 6 or an N-PASS pain score > 3.
3. Target Sedation: a N-PASS sedation score between −2 and −5, representing a moderate, clinically desirable level of sedation.

These binary outcomes (effective vs. ineffective analgesia; target vs. non-target sedation) were used in the generalized mixed-effect regression models to analyze changes over time and associations with fentanyl dosage.

### Statistical analysis

Descriptive statistics were computed for all analyzed variables: continuous variables were described as mean ± standard deviation (SD), whereas absolute frequencies and percentages were used for categorical variables. Statistical analyses were performed to evaluate the relationship between pain assessment scales and changes over time. To examine the correlation between pain scores, repeated measures correlation Pearson coefficient (r_rm) was estimated and reported with 95% confidence intervals (CI) and *p*-value. R-squared (R²) was also computed. Univariable generalized mixed-effect regression models were used to investigate the variations across observational periods of pain and sedation scores. Moreover, multivariable generalized mixed-effect models were used to assess the association between effective analgesia and time, adjusting for fentanyl dosage. To deal with repeated measures on the same infants, a random intercept term was included in all these models. Obtained results were reported in terms of mean differences (MD) or Odds ratios (OR), with 95% CI and *p*-values. Bland-Altman analysis for agreement was also constructed to visualize the limits of agreement between the two scales. The mean difference (bias) between N-PASS and EDIN scores and its 95% CI were calculated. The 95% limits of agreement (LoA; bias ± 1.96 SD of the differences) were derived to quantify the expected range of disagreement between the two instruments in the study population (23). Scores were also categorized into clinically relevant bins: no/mild pain (EDIN ≤ 6 & N-PASS ≤ 3) and moderate/severe pain (EDIN > 6 or N-PASS > 3). The observed agreement and Cohen's kappa coefficient (*κ*) with its 95% CI were calculated to assess the reliability of this clinical classification. The significance level alpha was set at 0.05.

All analyses were carried out using R version 4.3.2 (The R Foundation for Statistical Computing, Austria, Vienna, 2023).

## Results

Twenty infants were enrolled in the study (12 males and 8 females). The mean gestational age was 39.1 ± 1.4 weeks, and the mean birth weight was 3,420 ± 560 g. At enrollment, seventeen infants had moderate and three had severe encephalopathy. Two infants developed seizures and received phenobarbitone. Table 1 displays the administered fentanyl dose, as well as the EDIN and N-PASS scores, over the study period.

**Table 1:** NPASS scores, EDIN scores and fentanyl administered doses during study period.

Variable	Time	Estimate	Std. Error	95% Conf. Int.		*p*-value
N-PASS Pain	Mean Day 1	2,40	0,29	1,82	2,97	
	Mean difference between Day 2 and Day 1	−0,49	0,31	−1,10	0,12	0,121
	Mean difference between Day 3 and Day 1	−1,07	0,32	−1,69	−0,45	0,001
	Mean difference between Day 4 and Day 1	−0,78	0,47	−1,70	0,14	0,098
N-PASS Sedation	Mean Day 1	−4,22	0,46	−5,12	−3,33	
	Mean difference between Day 2 and Day 1	−0,60	0,36	−1,31	0,11	0,101
	Mean difference between Day 3 and Day 1	−1,08	0,36	−1,79	−0,37	0,003
	Mean difference between Day 4 and Day 1	−1,67	0,55	−2,74	−0,60	0,003
EDIN	Mean Day 1	2,98	0,42	2,17	3,80	
	Mean difference between Day 2 and Day 1	0,15	0,50	−0,83	1,13	0,761
	Mean difference between Day 3 and Day 1	−0,71	0,51	−1,70	0,28	0,162
	Mean difference between Day 4 and Day 1	−0,81	0,71	−2,21	0,59	0,260
Fentanyl dosage (mcg/kg/h)	Mean Day 1	1,42	0,08	1,27	1,57	
	Mean difference between Day 2 and Day 1	0,37	0,06	0,25	0,50	<0,001
	Mean difference between Day 3 and Day 1	0,36	0,06	0,24	0,49	<0,001
	Mean difference between Day 4 and Day 1	−0,25	0,09	−0,43	−0,08	0,005

Therapeutic hypothermia was maintained at 33.5 °C for 72 h (Days 1–3). Day 4 represents the period of controlled rewarming. Mean differences and their standard error were estimated through univariable linear mixed-effect regression models, which included a random intercept term to account for repeated measures on the same subjects, and time (days) as a fixed effect. Std. Error, Standard error; Conf. Int., Confidence interval.

**Figure 1:**
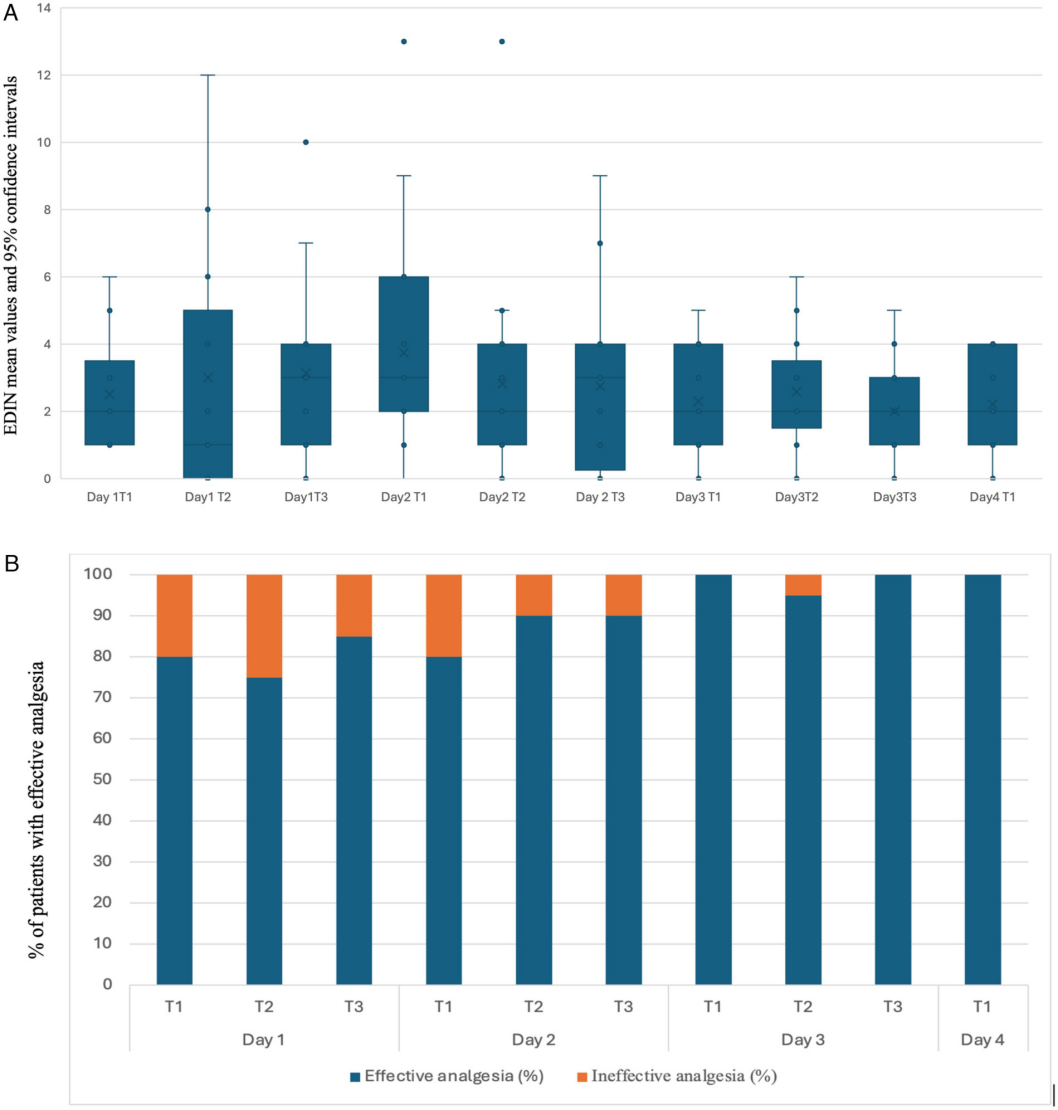
**(A)** EDIN score mean values and confidence intervals during the study period. The *Y* axis shows EDIN mean values and 95% interval of confidence. The EDIN score is assessed three times a day on days 1, 2, and 3 (T1, T2, T3) and once a day on day 4 during rewarming. **(B)** Effective analgesia assessed using the EDIN pain scale. The EDIN score is assessed three times a day on days 1, 2, and 3 (T1, T2, T3) and once a day on day 4 during rewarming. The *Y* axis shows % of patients with effective analgesia, as evaluated by EDIN score. Effective analgesia: EDIN score ≤ 6. Ineffective analgesia: EDIN score > 6.

**Figure 2:**
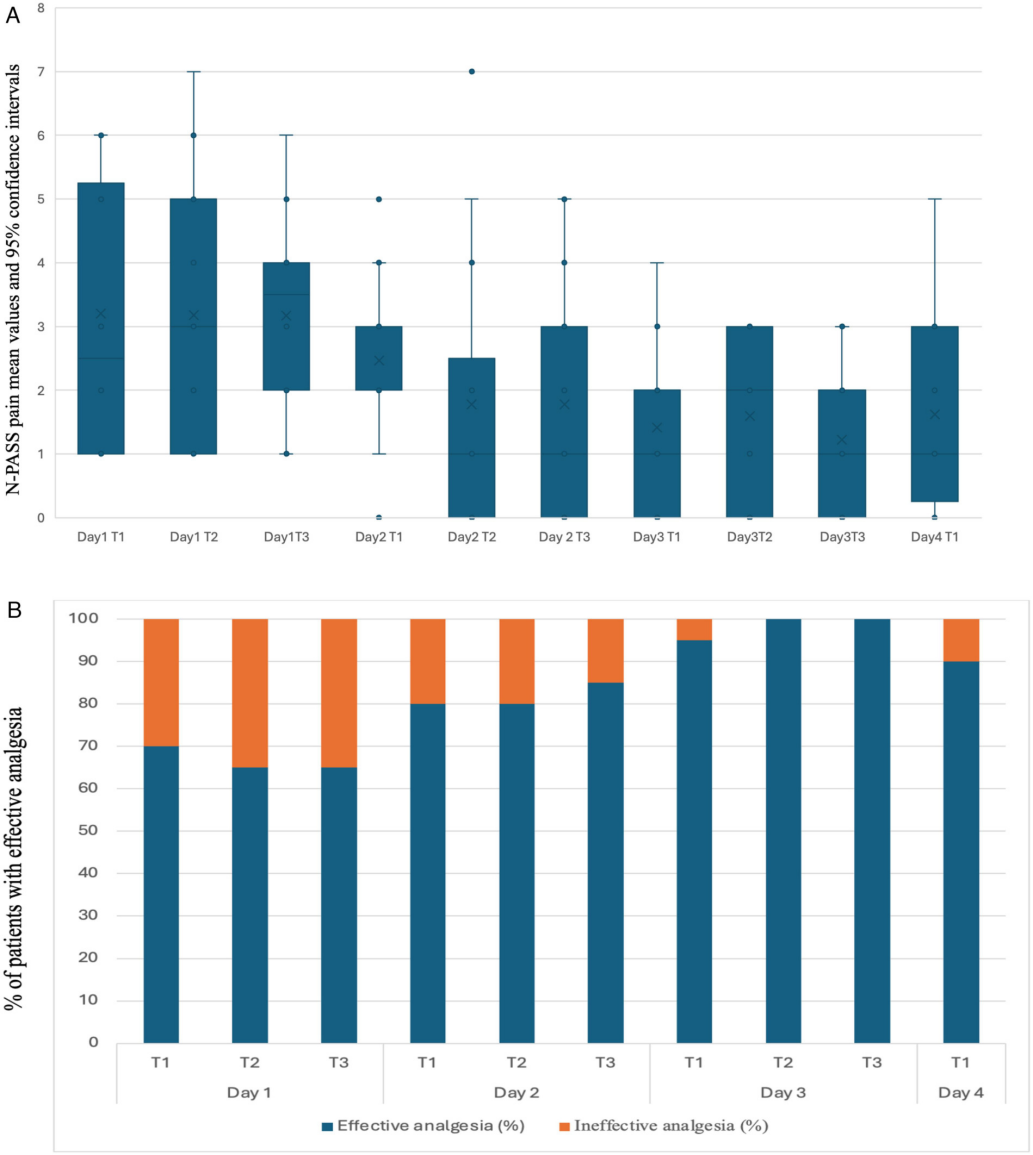
**(A)** N-PASS pain score mean values and confidence intervals during the study period. The N-PASS pain score is assessed three times a day on days 1, 2, and 3 (T1, T2, T3) and once a day on day 4 during rewarming. The *Y* axis shows N-PASS pain score mean values and 95% confidence intervals. **(B)** Effective analgesia assessed using the N-PASS pain scale. The N-PASS pain score is assessed three times a day on days 1, 2, and 3 (T1, T2, T3) and once a day on day 4 during rewarming. The *Y* axis shows % of patients with effective analgesia, as evaluated by N-PASS pain score. Effective analgesia: N-PASS pain score ≤ 3. Ineffective analgesia: N-PASS pain score > 6.

**Figure 3:**
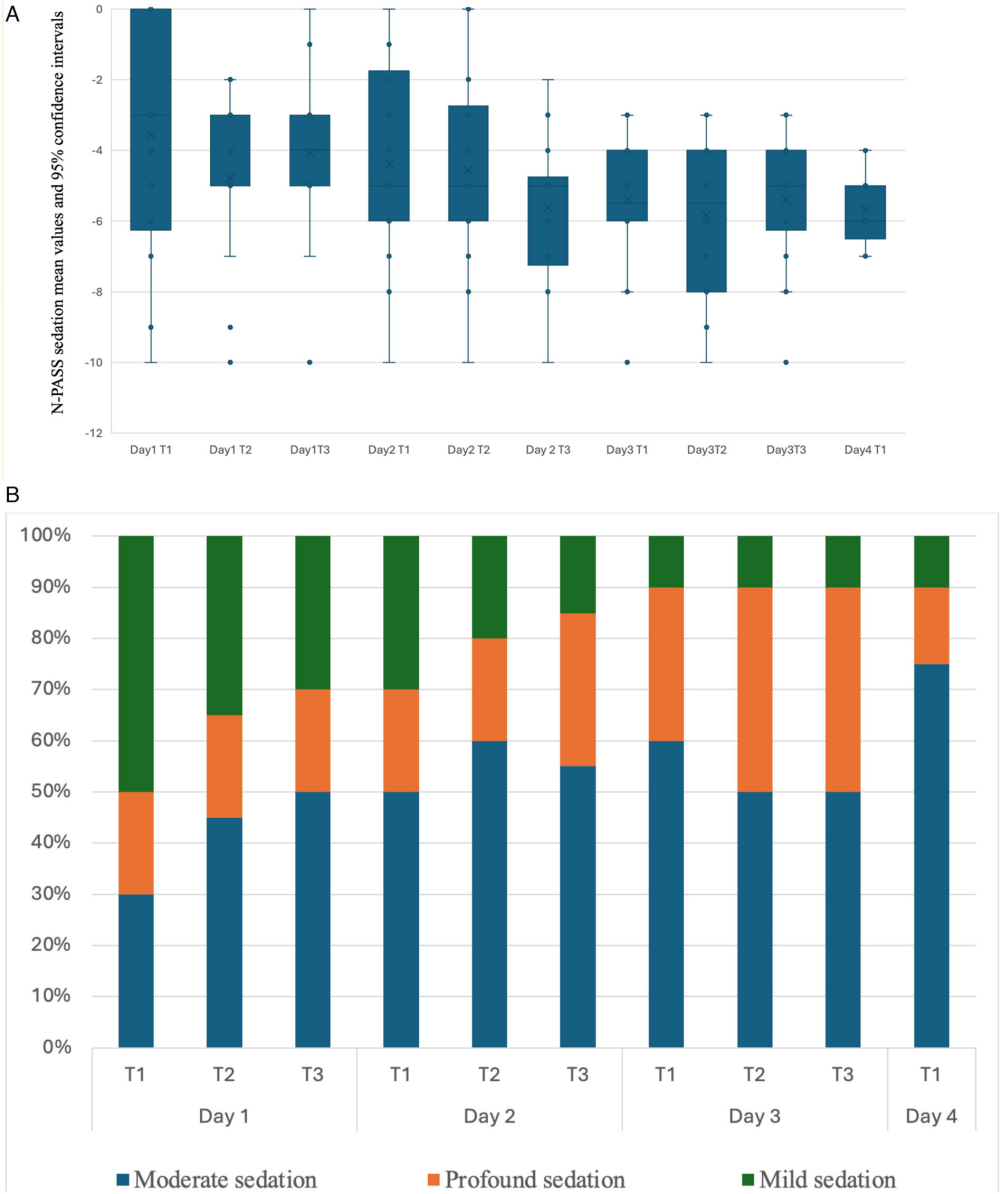
**(A)** N-PASS sedation mean values and confidence intervals during the study period. The N-PASS sedation score is assessed three times a day on days 1, 2, and 3 (T1, T2, T3) and once a day on day 4 during rewarming. The *Y* axis shows N-PASS pain score mean values and 95% confidence intervals. **(B)** Effective sedation assessed using the N-PASS sedation scale. The N-PASS sedation is assessed three times a day on days 1, 2, and 3 (T1, T2, T3) and once a day on day 4 during rewarming. The *Y* axis shows % of patients with effective analgesia, as evaluated by N-PASS sedation score. Moderate sedation: N-PASS sedation between −2 and −5. Profound: N-PASS sedation < −5. Mild sedation: N-PASS sedation > −2.

**Figure 4:**
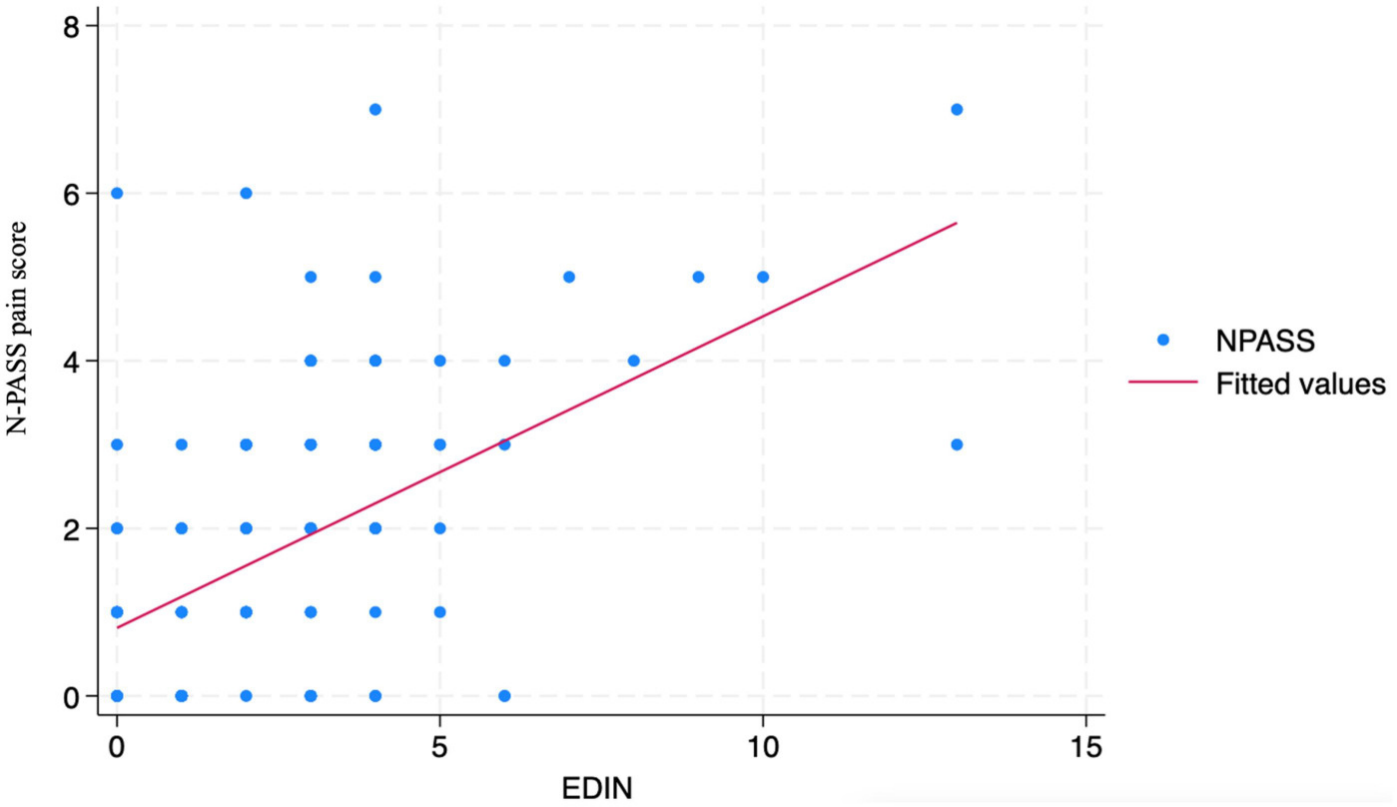
EDIN and N-PASS pain score correlation. The *y*-axis represents the N-PASS pain score. The *x*-axis represents EDIN score.r_rm = 0.409, 95% CI 0.242 to 0.552, *p* < 0.001.

Fentanyl dosage was escalated during the study period in accordance with the study protocol. The mean N-PASS pain and sedation scores were significantly lower on day 3 than on day 1, whereas EDIN scores did not differ significantly (Table 1). Figures 1A, 2A, 3A show mean values and 95%confidence intervals for EDIN, n-PASS pain and N-PASS sedation respectively. Figures 1B, 2B show effective analgesia, assessed using the EDIN and N-PASS pain scale, respectively, whereas Figure 3B shows effective sedation, assessed using the N-PASS sedation scale. Effective analgesia was associated with time for both EDIN (OR = 3.12; 95% CI 1.36–7.16; *p* = 0.007) and N-PASS pain (OR =  3.06; 95% CI 1.51–6.16; *p* = 0.002) scores. In contrast N-PASS sedation was not associated with time (OR = 1.41; 95% CI 0.97–2.04; *p* = 0.069). Changes over time in any of the scales were not associated with the administered fentanyl dosage. A significant correlation was found between EDIN and N-PASS pain scores (r_rm = 0.409, 95% CI 0.242–0.552, *p* < 0.001; R^2^ = 0.167) (Figure 4).

The Bland-Altman analysis revealed a mean difference (bias) of −0.61 (95% CI: −0.85 to −0.36), indicating that, on average, N-PASS scores were 0.61 points lower than EDIN scores**.** The 95% limits of agreement were wide, ranging from −5.87 to 4.65. This indicates that for an individual assessment, the N-PASS score could be as much as 5.87 points lower or 4.65 points higher than the EDIN score. When classifying pain states, the two scales showed an observed agreement of 72.1% for distinguishing no/mild vs. moderate/severe pain. However, Cohen's kappa was 0.32 (95% CI: 0.19–0.45), indicating only fair agreement beyond what would be expected by chance.

## Discussion

This study provides a comparative analysis of two neonatal pain assessment scales—the EDIN and N-PASS—in a cohort of asphyxiated newborns undergoing TH for HIE. Our findings demonstrate a statistically significant, moderate positive correlation between EDIN and N-PASS pain scores, indicating that these scales measure related, yet distinct, aspects of the pain experience in this vulnerable population. The observed correlation suggests that both tools are sensitive to changes in neonatal pain states during TH. However, the moderate strength of the correlation implies that EDIN and N-PASS may capture different dimensions of pain or may differ in their sensitivity to specific pain indicators under hypothermic conditions. The N-PASS scale, which incorporates both physiological and behavioral parameters, demonstrated a significant reduction in pain scores over the treatment period, aligning with escalating fentanyl administration. In contrast, EDIN scores, which focus exclusively on behavioral indicators of prolonged pain, remained relatively stable over time. This divergence may be attributed to the differential effects of TH and sedation on behavioral vs. composite (behavioral and physiological) pain responses. Hypothermia itself can suppress behavioral manifestations of pain, such as facial activity and body movements, which form the core of the EDIN scale (10–13). Conversely, N-PASS includes vital sign components that may remain responsive even under hypothermic and sedated conditions. The finding that effective analgesia, as measured by both scales, was associated significantly with time but not with the administered fentanyl dosage warrants careful consideration. This suggests that the natural evolution of HIE, rather than pharmacological intervention alone, may significantly affect pain expression during TH. As encephalopathy develops over time, neurological responsiveness may change, independently affecting pain scale measurements (10–13, 22, 24). While the natural evolution of HIE could be a primary driver of changing pain expression, a critical methodological factor must also be acknowledged. Our study, by design, reflected standard clinical protocol in which the fentanyl infusion was actively titrated in response to elevated pain scores (EDIN > 6 or N-PASS > 3). This creates a fundamental feedback loop: higher pain scores trigger dose increases, which, if effective, should subsequently lower the pain scores. In such a dynamic system, a simple cross-sectional or longitudinal association between the absolute dose and the concurrent score is inherently obscured. The administered dose at any point is a function of the prior pain state, not an independent variable. Therefore, the lack of a direct statistical association in our models does not imply that fentanyl was ineffective; rather, it reflects the reality of protocolized, responsive analgesia where the dose is continually adjusted to achieve a target outcome (pain score below threshold). This mechanistic complexity makes it difficult to isolate a classic pharmacokinetic/pharmacodynamic relationship from observational data in this setting. Future studies aiming to define optimal dosing might consider methodologies that can account for this temporal dependency, such as time-lagged analyses or modeling dose as a time-varying covariate with careful adjustment for the indication (pain score) (17, 22).

Nevertheless, in the specific context of TH-managed neonates, both scales offer distinct advantages. The N-PASS provides a comprehensive assessment by integrating physiological parameters with behavioral indicators, potentially offering a more complete picture of the neonate's state, particularly in sedated or neurologically depressed infants. Its bidirectional scoring system also allows simultaneous monitoring of both pain and sedation, which is particularly valuable in this population where both states commonly coexist and require careful balancing. The EDIN scale, developed specifically for assessing prolonged pain in neonates, may be particularly suited for detecting more subtle, persistent discomfort during the extended TH protocol. Its exclusive focus on behavioral indicators might make it less susceptible to confounding by physiological instability common in HIE patients. However, this same characteristic may also limit its sensitivity in severely encephalopathic infants who demonstrate minimal behavioral responses.

In addition, while EDIN and N-PASS scores showed moderate correlation (r_rm = 0.409), agreement analyses revealed they are not clinically interchangeable. The Bland-Altman plot demonstrated wide limits of agreement, indicating that for individual infants, scores could differ by nearly 6 points—a clinically significant discrepancy that could lead to different treatment decisions. Furthermore, categorical agreement for pain classification was only fair (*κ* = 0.32). This supports our interpretation that the scales capture different dimensions of the neonatal experience during TH. Notably, EDIN's sensitivity to behavioral manifestations may be significantly attenuated by hypothermia, whereas N-PASS—with its combined behavioral and physiological framework—provides a more robust and clinically actionable assessment in this unique therapeutic context. Further consideration emerges from the analysis of sedation. Despite the protocol targeting moderate sedation (N-PASS sedation score between −2 and −5), our data show that patients maintained an optimal sedation level in only 40%–50% of the cases. A temporal analysis revealed a distinct pattern: during the initial phase of therapeutic hypothermia, there was a higher prevalence of patients with N-PASS scores > −2, indicating insufficient sedation. Conversely, towards the end of the treatment period, a shift was observed towards N-PASS scores < −5, suggesting a tendency towards deeper sedation. This dynamic profile suggests that maintaining a stable and optimal sedative state during TH is particularly complex. The initial undersedation may be related to the acute stress response and evolving encephalopathy, while the later oversedation could reflect cumulative drug effects or altered pharmacodynamics during prolonged hypothermia and rewarming. This risk is heightened by the significantly reduced hepatic metabolism and renal clearance of fentanyl during sustained hypothermia. This pharmacokinetic alteration, a well-documented effect of cooling, leads to drug accumulation over the 72-hour period, increasing the risk of oversedation (25–27, 30). The pattern of sedation we observed—early undersedation followed by later oversedation—highlights the practical difficulty of maintaining a stable neuroactive drug effect throughout the dynamic phases of cooling. The failure to consistently achieve the sedation target throughout the therapeutic course highlights the need for even closer monitoring and potentially more dynamic, responsive, and personalized sedative titration protocols in this critical population. Our findings align closely with the observations reported by Üner et al., who similarly documented challenges in maintaining optimal N-PASS sedation scores throughout the course of therapeutic hypothermia, with a tendency toward undersedation early in treatment and oversedation during the later phases (17).

The clinical implications of this pattern are significant. Early undersedation may leave infants exposed to procedural and environmental stress, which can provoke detrimental sympathetic activation, leading to hemodynamic instability, increased cerebral metabolic demand, and potentially undermining the neuroprotective intent of hypothermia. Conversely, later oversedation is also concerning. Profound sedation can mask the clinical signs of evolving seizures, complicate neurological assessment, and may be associated with longer ventilator dependence. Furthermore, accumulating preclinical and clinical evidence suggests that prolonged or excessive exposure to sedatives like fentanyl in the developing brain may carry intrinsic neurotoxic risks or contribute to aberrant neurodevelopment (22, 28, 29). Therefore, the narrow target range for moderate sedation represents a critical balance between mitigating harmful stress and avoiding iatrogenic complications. Our data underscore the practical difficulty of maintaining this balance throughout the 72-hour cooling period and subsequent rewarming, pointing to a clear area for improved, personalized protocols.

Several limitations should be considered when interpreting these results. First, the single-center design and relatively small sample size limit the generalizability of our findings. Second, the blood level measurement of fentanyl was not considered because it was not among the objectives of the study. Furthermore, the potential for assessor bias, despite the use of validated scales, must also be acknowledged, as the nursing staff administering the scales were not blinded to the clinical context or the phase of treatment. Furthermore, the clinical profile of infants undergoing therapeutic hypothermia for HIE is notably complex and dynamic. In this cohort, two infants developed seizures requiring phenobarbital. We did not statistically adjust for these or other potential confounders—including ventilation status or hemodynamic instability requiring inotropic support—in our models. It is important to recognize that seizures, anticonvulsant therapy, and critical illness can independently affect levels of consciousness, motor tone, and physiological stability, which are core elements of both the EDIN and N-PASS scales. Consequently, the pain and sedation scores presented here represent a composite measure of the infant's clinical state, reflecting not only pain but also the effects of the underlying encephalopathy, concurrent interventions, and the physiological impact of cooling itself. This interpretive challenge is inherent to pain assessment in critically ill neonates and highlights the necessity of contextualizing scale scores within the broader clinical picture (2, 20, 22). Future research should focus on longitudinal assessments of pain scale performance throughout the cooling, rewarming, and recovery phases, as neurological status evolves. Correlation with neurophysiological measures, such as EEG patterns or neuroimaging findings, could provide additional validation. Studies comparing scale performance with biochemical stress markers may further elucidate their biological correlates in the context of TH, and incorporating plasma fentanyl levels could provide additional valuable insight. Despite the limitations of this study, based on our findings and clinical experience, although the two scales correlate, we consider N-PASS to be more suitable in the context of therapeutic hypothermia, for pain and sedation assessment. Its principal advantage lies in its multidimensional design, which integrates physiological parameters that remain responsive despite the behavioral suppression induced by cooling, thereby mitigating a key limitation of purely behavioral scales like EDIN. The clinical utility of N-PASS is further enhanced by its ability to provide a simultaneous, bidirectional evaluation of both analgesia and sedation on a single scale, facilitating more integrated clinical decision-making. Based on this evidence and our subsequent clinical experience, the N-PASS scale has been adopted as the standard assessment tool in our unit for infants undergoing TH.

## Conclusion

Both EDIN and N-PASS demonstrate utility for pain assessment in asphyxiated newborns undergoingTH, with a statistically significant correlation supporting their concurrent validity. However, their differential responsiveness over time and to interventions suggests they may capture complementary rather than identical constructs. The N-PASS's incorporation of physiological parameters and its ability to simultaneously assess analgesia and sedation make it particularly valuable in this complex population. Clinicians should recognize that scale selection may influence pain detection and management decisions, and understanding the unique characteristics of each tool is essential for optimal pain management in neonates with HIE receiving TH.

## Data Availability

The raw data supporting the conclusions of this article will be made available by the authors, without undue reservation.
