# Supplementary material for: EDIN and N-PASS pain scale comparison in asphyxiated newborns treated with therapeutic hypothermia
Source: Front Pain Res (Lausanne). 2026 Mar 23;7:1783611. doi: 10.3389/fpain.2026.1783611 (PMC13050844; doi:10.3389/fpain.2026.1783611)
Supplement: Supplementary file 1 [file Datasheet1.docx]

**Supplementary materials**

**Statistical analysis: sample size definition**

Based on prior neonatal pain scale validation studies comparing multidimensional and behavioral scales in critically ill populations, we anticipated a **moderate correlation** between EDIN and N-PASS. We conservatively set the expected population correlation coefficient (**ρ**) at **0.50**. Each infant was assessed three times daily. Accounting for potential missing data or early protocol discontinuation, we planned for a **minimum of 6 valid assessments per infant** over the 72-hour cooling and rewarming period. This results in **120 total data points** for the correlation analysis. The two-sided alpha was set at **0.05** and we targeted a conventional statistical power **(1 - β)** of **80%.** We anticipated a moderate within-subject correlation of repeated measures (ICC) of approximately **0.60,** reflecting that pain scores from the same infant are more similar to each other than to scores from different infants. The effective sample size (N_eff) in a repeated measures correlation can be approximated by accounting for the number of measurements (k) and the ICC. Using methods for power in multilevel/longitudinal designs focused on level-2 (between-subject) effects, the required number of clusters (subjects) is the primary determinant. Simulations and power calculations for two-level models (measurements nested within infants) with a continuous outcome at both levels indicate that with these parameters, **a sample size of 20 infants provides >80% power**to detect a between-subjects correlation of 0.50. A sample size of **N = 20 infants** was determined to be sufficient based on an a priori power analysis for the primary aim of establishing a significant correlation between two pain scales. This calculation accounts for the repeated measures design, which efficiently increases the information yield per subject. This sample size is also aligned with similar methodological studies comparing assessment tools in neonatal intensive care and is logistically feasible within a single-center study over a defined enrollment period.

**Supplementary tables**
